# Communicating health crisis: a content analysis of global media framing of COVID-19

**DOI:** 10.34172/hpp.2020.40

**Published:** 2020-07-12

**Authors:** Jude Nwakpoke Ogbodo, Emmanuel Chike Onwe, Joseph Chukwu, Chinedu Jude Nwasum, Ekwutosi Sanita Nwakpu, Simon Ugochukwu Nwankwo, Samuel Nwamini, Stephen Elem, Nelson Iroabuchi Ogbaeja

**Affiliations:** ^1^Department of Mass Communication, Ebonyi State University, Abakaliki, Nigeria; ^2^Oasis Research Institute, 35 Afkpo Road, Abakaliki, Nigeria; ^3^Department of Mass Communication, Alex-Ekwueme Federal University, Ndufu-Alike, Ikwo, Nigeria

**Keywords:** COVID-19, Coronavirus, Framing pandemic, Health crisis, Global media

## Abstract

**Background:** This study examines the global media framing of coronavirus disease 2019(COVID-19) to understand the dominant frames and how choice of words compares in the media. Periods of health crisis such as the outbreak of coronavirus pandemic add to the enormous burden of the media in keeping people constantly informed. Extant literature suggests that when a message is released through the media, what matters most is not what is said but how it is said. As such, the media could either mitigate or accentuate the crisis depending on the major frames adopted for the coverage.

**Methods:** The study utilises content analysis. Data were sourced from LexisNexis database and two websites that yielded 6145 items used for the analysis. Nine predetermined frames were used for the coding.

**Results:** Human Interest and fear/scaremongering frames dominated the global media coverage of the pandemic. We align our finding with the constructionist frame perspective which assumes that the media as information processor creates ‘interpretative packages’ in order to both reflect and add to the ‘issue culture’ because frames that paradigmatically dominate event coverage also dominate audience response. The language of the coverage of COVID-19 combines gloom, hope, precaution and frustration at varied proportions.

**Conclusion:** We conclude that global media coverage of COVID-19 was high, but the framing lacks coherence and sufficient self-efficacy and this can be associated with media’s obsession for breaking news. The preponderance of these frames not only shapes public perception and attitudes towards the pandemic but also risks causing more problems for those with existing health conditions due to fear or panic attack.

## Introduction

The world is battling the coronavirus disease 2019 (COVID-19) pandemic, which as of 20^th^ April 2020, 01:00 BST, has killed 155 124 with 2 285 210 confirmed cases in 213 countries around the globe.^1^ As the outbreak of the new coronavirus continues to evolve, it is difficult to arrive at the real death toll so far, especially because some countries were accused of covering up the actual number of victims or deaths related to the virus.^2^ Nonetheless, its spread has had enormous, far reaching global implications.

Whenever a health crisis such as the outbreak of COVID-19 pandemic emerges, the amount of information flow is overwhelming and this requires extra care in order to minimise the risk of the crisis. Thus, it is important to report the pandemic in a way that helps to douse the risk of the crisis rather than increase it.^3^ Literature has increasingly enriched the best practices about communicating health crisis,^3,4^although gaps are admitted. For example, a review by Glik^5^ established no standard set of competencies for public health crisis communication. The review concluded that this may have been the case because the discipline is still budding and/or because this area of research is a hybrid one whose literature is sparse.

As an institution saddled with the responsibility of disseminating information, mass media wields strong influence in shaping public opinion and decision making. Indeed, Schram^6^ argues that ‘‘news exists in the minds of men. It is not an event; it is something perceived after the event… it is an attempt to reconstruct the essential framework of the event which is calculated to make the event meaningful to the reader’’. This also aligns with the assertion that ‘‘when a message is emitted it is not only what is *said* that has a significance but also the *way* it is said, and what is *not said but could be said* .’’^7^ Therefore, the language of communicating the virus is essential for escalating or dowsing the tension caused by the pandemic.

### Contextualising COVID-19 in the media

The media’s inclusion or exclusion (inadvertently or otherwise) of some certain words in its coverage of the pandemic can be queried and often misinterpreted. Indeed, research acknowledges that even when the media role fails to confound the story, the reportage of issues such as public health crisis regularly stimulates blames for over reporting, inadequate or inaccurate coverage.^8-10^ This has been the case for different coverage of health crises such as Anthrax and H151.^4,8^ So far, the global media coverage of COVID-19 has not shown any exception.

Nonetheless, the shortcomings in the reportage of the evolving COVID-19 pandemic could potentially trigger more global health concerns. For instance, the nature of media framing of the pandemic may have accentuated public’s fear or lack of it as it were in 2001 when Anthrax health crisis was reported.^8^ Similarly, when President Trump tweeted that chloroquine and other related malaria drugs were useful for treating COVID-19, its framing in the media engineered panic buying of the drug in different countries. *The Punch* reported that hospitals in Nigeria recorded increased cases of chloroquine overdose as a result of Trump’s endorsement.^11^ The World Health Organization frowned at this and urged the media to feed the public with only the reports that have medical/scientific evidence.^12^ It issued a statement saying that ‘‘Hydroxychloroquine and chloroquine are under investigation in clinical trials for pre-exposure or post-exposure prophylaxis of SARS-CoV-2 infection, and treatment of patients with mild, moderate, and severe COVID-19’’.^12^ Therefore, vital information illuminating or addressing the COVID-19 health crisis may have long-term implications for public health.

### Framing COVID-19 health crises

During a period of pandemic, the ability of health bodies like WHO and CDC to connect with the public through the media may mean the difference between morbidity and mortality.^13^ How such organisations depict such issue is particularly impactful especially in the early days of the outbreak.^14^ Through framing, the media connects with the masses by telling the crisis story.^15^ Framing acknowledges the ability of the media message to define a situation and set the issue in motion for a debate.^16^ In connection with communicating the health crisis caused by COVID-19, framing allows us to identify how the media approaches the reportage of the pandemic and the preponderant frames that will in turn help to determine the effectiveness or otherwise of the coverage.^14^

Chiefly beginning with Iyengar’s seminal research^17^ on media framing of poverty, crime, and employment, researchers have classified some types of frames used by the media to pass their message across. Taking a cue from this pioneering research, Semetko and Valkenburg^18^ identified five generic frames – *conflict, human interest, economic consequences, morality* and*responsibility* that occur in news reports. A follow up research by Ogbodo^19^ advanced these frames and added *ethnicisation* and *politicisation* to make the generic frames seven. In this study, we further modify these frames by Semetko & Valkenburg^18^ and Ogbodo^19^ by adding two emerging frames (fear/scaremongering, and hope) employed in the reportage of the pandemic.

Therefore, as earlier hinted, the problem that necessitated this study is that the emergence of COVID-19 has been shrouded in speculative literature with minimal empirical understanding of how the media framing of the health crisis would help in mitigating or escalating it. People’s perception and interpretation of the ‘dreaded’ coronavirus would naturally stem from the way the virus is framed in the media. It is important that the media does not blow a critical issue like COVID-19 out of proportion to avoid creating unnecessary panic in the people, which is capable of leading to more health complications as the case may be. Engaging in constructive coverage of the pandemic would help the public to cope with the fear of the pandemic.

Reliant on the foregoing review, our study is interested in understanding the dominant frames adopted by global media to report COVID-19. In addition to that we want to know how language of covering COVID-19 compares in global media. No known research has focused on understanding the dominant frames employed by the global media to report the pandemic. Moreover, existing research has ignored a focus on how language or diction compares in the global media. Indeed, literature search reveals that most of the existing studies are anecdotal and relied on speculations to discuss the coronavirus outbreak without empirical proofs. This leaves a gap in literature. Our research empirically intervenes in this instance. Therefore, as part of the attempt to understand the dominant frames, we are measuring Economic consequences, Morality/Religious frame, Human Interest, Conflict, and Attribution of responsibility; Politicisation, Ethnicisation; Fear/scaremongering, and Hope frames. These will help us to understand what dominated the global media’s reportage of the COVID-19 pandemic (see Table 1).

## Material and Methods

### Timeframe

We begin our analysis from 29^th^ December 2019 when the first case of coronavirus was reported in Wuhan, China to 29^th^ April 2020, when the pandemic peaked across the world. Although COVID-19 pandemic is still evolving, we believe that the timeframe is long enough to elicit requisite information to guide our understanding of the ways global media frames the pandemic.

### Data collection and sampling

This study utilises content analysis, a technique which is systematically and objectively used to describe the apparent content of communication as well as draw inferences as the case may be. In so doing, our study systematically focuses on eight leading global media outfits in the Americas, *News York Times* and *CNN* ; Africa, *The Punch* ; Asia, *People’s Daily;* and Europe*, BBC, Daily Mail, Vatican News* and *Le Monde* . These eight media organisations are both elite, religious and popular and have global reach in this era of social media. We made sure that each continent has at least one media outfit, taking note of the continental severity in our choices. Factor such as the country with increasing number of COVID-19 deaths was also considered in the selection process. Grounded on these criteria, the authors believe that coverage in these media organisations provides a good context for understanding the framing of the pandemic and the language of coverage of the pandemic.

For all media outfits except *The Punch* , and *People’s Daily* , all reports containing the terms ‘‘Coronavirus OR COVID-19’’ were accessed from the LexisNexis General News database. *The Punch* and *People’s Daily* stories containing ‘‘Coronavirus OR COVID-19’’ were accessed from their websites. All articles were saved electronically, and subsequently printed and archived according to country and publication date. Although our search originally yielded 15 523 items on COVID-19, we decided to trim the number by removing stories that made just a passing reference to the pandemic as well as sponsored stories or advertorials.

To arrive at the sample size for the analysis, we considered Neuendorf’s^29^ aim of lessening standard errors for calculating conﬁdence interval. We further employed the consecutive days sampling format since the study period is just four months. Research has demonstrated that small samples (e.g., 5%-10%) arrived at by sampling successive days of coverage are sufficient enough to understand news contents over some times.^30^ The consecutive day sampling strategy varies systematically according to the amount of news and advertising space. For instance, a close observation reveals that Sunday issues seem to make more spaces available for news unlike Saturday’s. Reliant on this conviction, our sample for this study was constructed by randomly picking relevant stories from every day of the study period. Given that the amount of the items for the analysis was still high, we resorted to sampling 20% of news items per day. Stories that made a passing reference to the virus were automatically eliminated. This left us with 6145 items for the analysis.

### Coder agreement

Within us, we assigned two coders the responsibility of coding the variables while monitoring their progress to enhance reliability. In order to ensure quality outcomes, the two coders proceeded to code 100 randomly selected items. This helped to establish inter-coder reliability. Coder agreement rates range from 85% or more for every variable analysed. This was done using Neuendorf’s PRAM computer programme.^29^

## Results

As earlier hinted, sources from LexisNexis database and sources from two websites (https://punchng.com & http://www.peopledaily.com.cn) yielded a total of 6145 items (news, reports, features and in-depth analysis) used for the study. As Table 2 demonstrates, out of 6145 analysed items, *BBC* had a total of 1023 (16.6%), *Daily Mail* had 1124 (18.3%), *Le Monde* had 347 (5.6%), *CNN* had 1041 (16.9%), *NYT* had 982 (15.9), *The Punch* had 458 (7.5) while *People’s Daily* had 737 (11.9). This suggests that *Daily Mail, CNN, BBC* and *NYT* had more relevant coverage of the pandemic. The four media outlets account for 67.7% the entire coverage. The *People’s Daily* , which prides itself as the largest newspaper group in China is fifth in the ranking with 11.9% of the coverage.

### Human interest 

**Figure 1:**
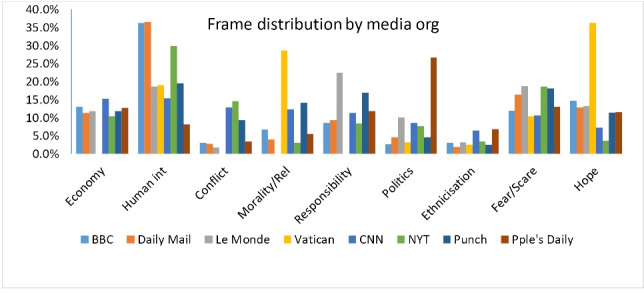
Frame distribution showing the proportions for each media outfit.

**Figure 2:**
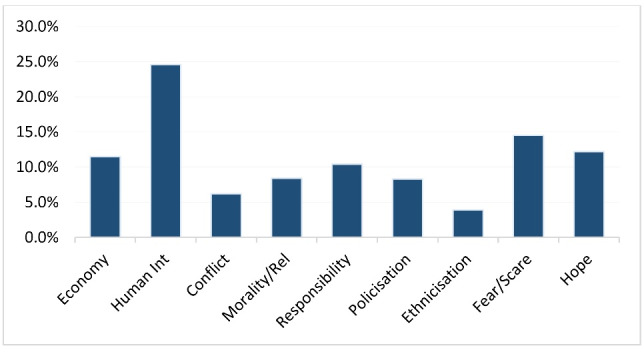
Combined frame distribution showing the proportions for each media outfit.

As Figures 1 and 2 demonstrate, the dominant frame employed by the media to report the novel coronavirus is *human interest* . It accounts for 24.6% of the entire stories analysed for the study. Instances of this frame that draw on the impact of the pandemic on human beings across the world abound. For instance, ‘‘…Confinement, illness and stagnation weigh heavily on the public already staggering under the health crisis. Thousands of stores, workshops and large companies have stopped functioning, and many people have exhausted their resources and are struggling’’.^31^ This story captures the gory tales of the effects of COVID-19 on Iranians whose case is worsened by the numerous sanctions imposed by the United States and its allies, making it hard to import even medical supplies from overseas.

Another example of human interest frame is the impact of the pandemic as ‘‘16.8 million jobless claims were filed over a 3-week period in the US’’.^32^ The jobless situation triggered by the lockdown which became necessary to check the spread of the novel coronavirus has also left in its trail, a high demand for benefits to cushion the effects of the pandemic. Another relevant story is that of ‘‘New York governor says coronavirus has killed 4000 more people than 9/11. ..9/11 was supposed to be the darkest day in New York for a generation,”^33^ Cuomo said in a press briefing. “We lost 2753 lives on 9/11. We’ve lost over 7000 lives to this crisis. That is so shocking and painful and breath-taking, I don’t even have the words for it”.^33^ This story episodically relates the unfortunate 9/11 terrorist attack to the present situation caused by COVID-19.

Still on human interest frame, the debilitating situation of Rohingya refugees in Bangladesh calls for pity. Thus, ‘‘Cox’s Bazar, home to the world’s largest refugee camp, is under lockdown… in an attempt to prevent the spread of coronavirus from reaching the sprawling and crowded refugee camps that house about 1 million Rohingya people’’.^34^ In the stories ‘‘Wuhan, center of coronavirus outbreak, is being cut off by Chinese authorities’’^35^; and ‘‘Coronavirus lockdowns torment an army of poor migrant workers in China’’^36^ further paint the gory picture of the impact of the virus on the welfare of the people. All the media employed this frame more frequently, an indication that the impact of the pandemic is global in scale and far-reaching. Another touching story of the pandemic is contained in the *Vatican News* wherein, it captioned it ‘‘Homeless during Covid-19’’. The story captures the ordeals of one ‘‘Debbie, a homeless woman living in Boston during Covid-19’.^37^ The story elicits pity and it emphasises the devastating impact of the pandemic on the people.

Within the human interest frame, we extracted both the positive and negative impacts. *Daily Mail* had more cases of negatives frames in human interest, regularly reporting the death toll more often than the recovery. BBC was also an alarmist in this regard and played more roles in reporting the death tolls than the degree of victims who recovered from the virus. This may have accentuated fears in the public and may potentially lead to stress disorders. A similar case scenario was enacted during the outbreak of H1N1.^4^ Therefore the tendency to foreground only the negative human impact of the pandemic leaves more psychological impacts on the people and may be counterproductive in the fight against COVID-19.

### Fear/Scaremongering 

The second dominant frame is fear/scaremongering. Fear can, sometimes kill faster than the pandemic. The overwhelming nature of the virus has been hyped in the media and fear has gripped the people, some of who may have died or lived in apprehension. For instance, *CNN* reports that ‘‘Chinese President Xi Jinping has warned against the risk of a second wave of infections in the country as the global pandemic continues to spread’’.^27^ This has further aggravated the people’s fear. The issue is not that what the media reports is not true in this case, but overhyping it leads to more health or stressful conditions. The *BBC* also reports a high rise in the number of panic attack in the UK due to the fear and anxiety propagated via different media platforms. Thus, ‘‘you wake up, switch on the news, scroll through social media and see endless posts about coronavirus. Suddenly you’ve got a headache and your throat begins to feel dry. The symptoms are there when you’re worried but disappear when you’re distracted’’.^38^ In the same story, 25 year-old Lucy noted that the first thing she hears at her work place is her ‘‘colleagues talking about how coronavirus is affecting everyone. [Sequel to that] ‘I feel ill after coronavirus chat’’’. Another source in the story said, ‘‘if there wasn’t a hype about virus looming I definitely wouldn’t be as worried about these symptoms, I’d probably even dismiss them entirely’’.^38^ Some pieces of advice cited by *BBC* in the report is that people should stop viewing, reading or listening to news stories capable of triggering such anxious or distressed mood. Instead, they should only select what to view, read or listen to in order to shield themselves against such feelings.^38^

Although the media may inadvertently cause fear, it is important that it tones down on its way of reporting crisis to avoid overreaction in the process. In the story, ‘‘Britain will not meet its goal of full ventilator capacity by the time the coronavirus peaks’’, Britain’s Health Secretary, Matt Hancock was quoted as saying that ‘‘the country was on track to have ventilators for 18 000 patients – but they may not be in place in time’’.^39^ This puts people (the sufferers and the public) into fear of losing more lives.

Another implication of the high rise in media-induced fear is evident in the *CNN*^40^ report that ‘‘more Americans are turning to anti-anxiety medications as the coronavirus crisis has upended everyday life. Prescriptions for anti-anxiety medicine started climbing in mid-February, spiking 34% by March 15… The recent increase in usage was nearly twice as high for women, whose prescriptions jumped almost 40%, compared with men, who saw a 22.7% rise. It’s a sharp reversal from the pattern over the last five years, during which the use of drugs known as benzodiazepines — including Xanax, Klonopin, Ativan and Valium —declined 12.1%’’.^40^ This further portrays the dangers of media’s overreaction in the coverage of the virus and its attendant consequences on public health. Indeed, research has warned that ‘infodemic’, a new concept for explaining the excess information (real and fake) flow spreads faster than the pandemic itself.^41^

### Hope frame

Amidst the jeers and fears caused by the framing of the novel coronavirus, there are still cases of hope in fighting the pandemic. Hope is an essential counter measure that soothes the public even in a clearly overwhelming situation of COVID-19 health crisis. During the Haitian earthquake, Muralidharan et al^42^ report that the media coverage which emphasised hope in the devastating situation minimised the effects of the earthquakes on the people. In this study, hope frame ranks third and accounts for 12.20% of the entire stories used for the analysis.

For instance, ‘‘Spain’s rate of new coronavirus infections falls to its lowest yet for a second day in a row in latest sign the country is emerging from the worst of the pandemic’’.^43^ The language employed here expresses hope and reassurance to the people who have been bombarded with infodemic and hopeless reports portraying the virus as the worst thing that has happened to the world. Again, the hope frame is the foundation of the story ‘‘we place our hope in the Cross of Jesus, through which ‘we have been healed and embraced so that nothing and no one can separate us from His redeeming love.”^44^ Despite the effects of the pandemic on the masses and the global economy, God is the hope of believers.

The *CNN* ’s reportalso offers a glimpse of hope in dealing with the aftermath of the pandemic. For example, in one of its editorials, *CNN* writes that‘‘**…**in all likelihood, hope is not lost. “We tend to overestimate the likelihood of something happening, and we tend to underestimate our capacity to deal with it”.^45^ Similarly, another *NYT* story employing hope frame is ‘‘optimism is less distant as global coronavirus battle rages on’’.^46^ The Nigeria Centre for Disease Control was also quoted as saying that ‘‘Coronavirus will be defeated like Ebola in Nigeria.’’^47^

Another instance of hope frame is the story on *BBC* ‘‘Coronavirus: The good that can come out of an upside-down world’’.^28^ In the story, Matthew Syed writes that ‘‘our world has changed immensely in the last few weeks but amid the upheaval and distress, there are reasons to believe we can emerge from the crisis with some human qualities enhanced… But amid the darkness, there are also opportunities. Opportunities to reimagine the world and one’s place within it’’.^28^

### Economic consequences

In the coding process, we coded the economic frame into gain and loss sub-frames. The gain sub-frames focuses on the reports suggesting economic benefits or stimulus package meant to cushion the effects of the pandemic on the economy. The loss economic consequence sub-frame is contained in the stories that predominantly report the negative economic impacts of the crisis. For instance, in the report, ‘‘Economic growth in sub-Saharan Africa is forecast to fall sharply in 2020 and the region will suffer its first recession in 25 years, according to a World Bank report. [The report^48^ estimates that] COVID-19 will cost the region between $37 billion and $79 billion in output losses for 2020 due to a combination of effects including trade disruption and reduced remittances, tourism and foreign aid’’. The economic loss associated with the novel coronavirus is estimated to have a devastating economic consequences on the sub-Saharan Africa. On the positive economic note, *CNN* also reports that ‘‘The Fed just unleashed another $2.3 trillion to support the economy. The Federal Reserve is continuing its extraordinary efforts to prop up the US economy in the wake of the coronavirus pandemic’’.^49^ In another negative note, the *NYT* reports that ‘‘U.S. stocks plunge as coronavirus crisis spreads’’.^50^ This frame emphasises the economic downturn triggered by COVID-19 outbreak.

A report by Tappe of *CNN* expresses optimism about the economic bounce-back from the pandemic. Thus, ‘‘Fed chairman expects a “robust” economic recovery’’.^51^ The report quotes the Federal Reserve chairman Jerome Powell as saying that the ‘‘US economy should rebound fairly quickly when businesses reopen after the coronavirus lockdown ends… This is what the great fiscal power of the US is for, to protect the people the best we can from the hardships they’re facing. At the Fed, we do all we can to shepherd the economy through this difficult time”.^51^

### Ethnicisation

In the course of reporting the pandemic, the media may be emphasising stories that stimulate or deepen racial divides. For example, ‘‘French doctors are accused of racism and ‘treating Africans like human guinea pigs after saying Covid-19 vaccines should be trialled there…They said Africa has ‘no masks, treatments and no ICU’ for coronavirus patients’’.^26^ The idea of testing the vaccine in Africa has been widely condemned across the continent and this has been interpreted from an ethnic and racist prism. Recall that top African football legends like Didier Drogba and Samuel Eto condemned the remarks by the French physios as ‘deeply racist’.^26^

In the Vatican News report ‘‘Covid-19: U.S. Bishops warn against patient discrimination’’, United States Conference of Catholic Bishops (USCCB) issued a joint statement calling for ‘‘equal access to health services without discrimination during the Coronavirus pandemic’’.^52^ The statement continues ‘‘In many countries, the lack of respirators and intensive care units is forcing doctors and nurses to face dramatic choices between who to treat – and who not…Any discrimination on the basis of age or disability is ethically unacceptable…this is not a time to side-line our ethical and moral principles. It is a time to uphold them ever more strongly...Good and just stewardship of resources cannot include ignoring those on the periphery of society, but must serve the common good of all, without categorically excluding people based on ability, financial resources, age, immigration status, or race”.^52^

### Attribution of responsibility 

Attribution of responsibility was also varyingly employed in communicating COVID-19. Perhaps, by coincidence, Corona beer was regarded as the cause of the virus. In ‘‘Corona beer suspends production due to coronavirus’’, *Daily Mail*^53^ reports that ‘‘brewer of Corona beer has suspended production because of the coronaviruspandemic. The brand of lager - whose unfortunate name has made it a punchline during the health crisis - will not be produced after Mexico deemed it non-essential. Fears of a shortage have prompted panic-buying, with pictures from Mexican shops showing trolleys piled up with beer.^53^

The blame game for the spread of the virus across the UK is vividly captured in the story by *Le Monde* . ‘‘…Britain became an international anomaly: schools remained open. Politicians claimed to be creating ‘herd immunity’ by waiting for the ‘right moment’ before even asking citizens to distance or isolate themselves. Mass public events went ahead — the Cheltenham Festival races, et cetera — against the backdrop of a nation panic-buying groceries and toilet paper. Surely these desperate shoppers were a sign of the public’s lack of confidence in their prime minister, who seemed to be hiding behind the stature of his chief scientist and chief medical officer’’.^54^ The story blames leadership complacent for the spread of the virus in the UK. It suggests that the government of the UK failed to deal with the spread of the virus early enough hence the spread.

Another use of this frame pattern is evident in the story: ‘Did coronavirus leak from a research lab in Wuhan? Startling new theory is ‘no longer being discounted’ amid claims staff ‘got infected after being sprayed with blood’… Senior sources in the British government say that while ‘the balance of scientific advice’ is still that the deadly virus was first transmitted to humans from a live animal market in Wuhan, a leak from a laboratory in the Chinese city is ‘no longer being discounted’. The paper reports that ‘‘The state-run *People’s Daily* newspaper said in 2018 that it was ‘capable of conducting experiments with highly pathogenic microorganisms’ such as the deadly Ebola virus’’.^55^ This does not only confound the myth surrounding the emergence of the virus, but also adds to the attribution of responsibility and China has been accused of doing too little to contain the virus at the onset.

Attribution of responsibility is also inherent in the story that associates 5G technology to the spreading of the virus. For instance, ‘‘Scientists slam celebrities such as Woody Harrelson, Calum Best and Lee Ryan for ‘fanning the flames’ of false conspiracy theory blaming 5G technology for coronavirus… 5G masts have been set on fire around Britain after theories about the link between the mobile technology and Covid-19 circulated online’’.^56^ The 5G-COVID-19 connection has been widely shared on social media as well.

### Morality/Religion

Morality/Religion frame was also widely employed in the coverage of the crisis. For example, *NYT* reports that ‘‘in a pandemic, religion can be a balm and a risk’’.^57^ The story draws the implication of using religion to flout the social distancing guideline for checking the spread of the virus, especially in the Middle East where people held sway to their religion despite dangers of not maintaining social distance.^57^

A follow up story by the newspaper suggests that ‘‘Virus soars among ultra-orthodox Jews as many flout Israel’s rules’’.^58^ Ultra-religious Jews were considered a threat to the fight against the pandemic because of their strong faith. A *BBC* report tagged ‘‘Is coronavirus coming between people and their faith?’’^23^ also tries to draw our attention to the new normal in the changing patterns of worshipping in different religions. Thus, ‘‘as concern over the spread of coronavirus grows, people around the world are changing the way they dothings… Churches, mosques, temples and synagogues are also changing rituals in an effort to contain the spread of the virus’’.^23^ Sequel to that, ‘‘Catholic churches from Ghana to the US and Europe have changed the way they carry out Mass in an effort to stop infection. Priests now place the wafer in people’s hands rather than on their tongues, and have stopped giving wine in the communal chalice’’.^23^

Indeed, the outbreak of COVID-19 has come between people and their religion. Many patterns of religious and spiritual life of worshipers around the world are adjusting rapidly in line with the prevention guidelines for the virus. Otherwise, Lebo Diseko of *BBC* queries ‘‘how do you tell people not to hug a grieving widow at a funeral?’’ Rabbi Jackie Tabick, from London’s West Central Liberal Synagogue responded thus: “It’s a really tough one. I think I’m going to say something like: ‘I know that everybody wants to physically express their love for the widow, but really the best way that you can express your love these days - and I know she will understand too - is to talk to her, nod to her, but don’t touch, because it’s really not the right thing to do at the moment”.^23^

Another related report by the *Vatican News* also employed religious frame. Thus, ‘‘The President of Latin American Bishops’ Council (known as “CELAM” from its initials in Spanish), Archbishop Miguel Cabrejos Vidarte of Trujillo, Peru, appeals for generous, compassionate and merciful love for the most needy who are suffering as a result of the Covid-19 pandemic’’.^44^ The report continues, “In these circumstances,” Archbishop Cabrejos writes, “a true love for one’s neighbor and for the care of the most vulnerable people is shown in respect for the measures linked to physical and social isolation, but these restrictions should not lead us to erect walls and barriers in our hearts”.^44^ This story therefore brings out the importance of love and compassionate living in fighting the overwhelming pandemic.

### Politicisation of the pandemic

Amidst the fight against the COVID-19, politics of the pandemic has pitted political parties against each other and this has resulted in difficult decisions, actions and inactions that have been interpreted from the political lens. For instance, the political rift between Washington and Tehran has intensified as the pandemic rages on. This rift accounts for why US sanctions against Iran has prevented them from buying medical supplies to deal with the scourge. Nonetheless, *Le Monde*^31^ reports that ‘‘US President Donald Trump has offered humanitarian aid via Switzerland — but without any concrete proposal (he says, ‘All they have to do is ask’). Iranian President Hassan Rohani responded to this on 4 March, saying that if the US truly wanted to help Iran combat the virus, it should lift sanctions on the country, including its prohibition on importing medical supplies. ..They’ve appeared with a mask of sympathy that “we also want to help the people of Iran”.^31^

The politics of the pandemic also extends to the report that partly reads ‘‘unless protests, actions, political parties, peoples and states change the script. Many say ‘Politics doesn’t concern me’, until the day they realise it is *political* choices that force doctors to decide which patients live or die. That day has come. It is worse in central Europe, the Balkans and Africa, whose medical professionals have for years moved to safer countries where they are better paid; the situation there too is a result of *political* choices’’.^59^

In his contribution in *Le Monde* , Serge Halimi explains that ‘‘Protectionism, environmentalism, social justice and public health have come together. They are key elements of an anti-capitalist political coalition that is powerful enough to impose a programme of breaks’’.^59^ International politics of the pandemic also resulted in the spar between Washington and Beijing. For instance, in *CNN* ’s report^25^ ‘‘Chinese government after US criticism: “Accusations won’t get rid of the virus” “Smearing and accusations won’t get rid of the virus,” said Chinese Foreign Ministry spokesperson Zhao Lijian at a press conference in Beijing. “We hope to see the American people win its fight against the outbreak as early as possible…Zhao refrained from naming Pompeo or Trump in his rebuke, but added that “we hope that the American people will reject certain politicians’ actions to politicize the pandemic, stigmatize China, shift public attention and deflect blame.”^25^ Although President Trump has since toned down on his word war against the Chinese government for the handling of the pandemic at the onset, the politics of supremacy between the two world powers has been reignited by the fallout of the pandemic.

Politicisation frame is also inherent in the report, ‘‘Colorado Democrat claims Trump awarded ventilators as political favor to vulnerable senator’’.^60^ In thereport, a veteran Colorado Democrat Diana DeGette claimed in an interview with the *CNN* that President Donald Trump’s announcement that he would send 100 ventilators to Colorado ‘‘smacks of a political favour to vulnerable Republican senator Cory Gardner, after the federal government had not fulfilled the delegation’s request for the devices’’.^60^ The politics of the pandemic has been more eminent in the US as Democrats and Republicans jostle to make political gains out of the crisis ahead of the November Presidential election.

### Conflict frame 

The pandemic is already treated as a crisis across the world and the military, medical professionals, paramedics and journalists are working closely as ‘frontline’ personnel as they would during wars and other international crisis. Indeed, a New York ICU nurse noted that ‘‘battling coronavirus feels “like a war”.^22^ It is a challenging period and the media is braving the pandemic to bring an up-to-date information about the virus. In the conventional sense, COVID-19 has sparked political and economic conflicts between countries and within some countries. Examples of this frame include where President Donald Trump severally referred to the virus as an ‘invisible enemy’^61^ and the media has also adopted this concept to describe the coronavirus crisis. For instance, *BBC* reports that Trump has urged all US citizens to stay indoors in order to fight an ‘invisible enemy’.^62^ President Emmanuel Macron of France also used conflict terms such as ‘we are at war’ to describe the ‘invisible elusive enemy’.^63^

Thus, the pandemic expands the dimension of international rift over the handling of the pandemic in the early periods of the outbreak. In the story ‘‘Did the Chinese Government deliberately export Covid-19 to the rest of the world?’’^64^* People’s Daily* reports that ‘‘US President Donald Trump said at a news briefing that Beijing should face consequences if it was “knowingly responsible” for the coronavirus pandemic’’.^64^ In whatever way it is considered, COVID-19 is not just a health crisis, it has heightened international conflict between the China and the US and indeed the rest of the world and the media is complicit in the rift.

### Language of the coverage

Thelanguage of the coverage of the pandemic combines gloom, hope, precaution, anger and frustration at varied proportions.For instance, in the *Daily Mail* report‘‘The Queen addressed the UK in a poignant and historic television address amid coronavirus outbreak…urged Brits to remain resolute and assured millions watching the nation will pull together and ‘overcome’’.^65^ The reportsconcludes with **‘‘**we will meet again’’ to connect Brits with the war song rendered for the British soldiers during the WWII. The reports describes the Queen’s speech as rousing’’.^65^ Using such languages is good for motivating those in the frontline to enable them feel loved and cherished for the job they do.

The media used a mixture of mild and harsh words to report the pandemic. While the Western media predominantly employed terms that clearly suggest their freedom of speech, they do not engage in the adulation of leaders of their countries but report issue the way they appear.In one of its reports for instance, *NYT* used ‘Apocalyptic’^66^to describe the coronavirus surge in New York. *The Punch* also had some strong words for the virus. It occasionally described it as the ‘raging Coronavirus monster’.^67^ In one of its editorials, *People’s Daily* employed rancorous terms to counter narratives from the Western media. Thus, ‘‘as the COVID-19 pandemic continues to worsen in western nations, some scholars and experts have pointed fingers at the pandemic control measures China took during the early stages of the outbreak, believing that it’s China’s fault the lethal virus is now rampant around the globe.^64^ [This is]…from the fertile imaginations of obscure conspiracy theorists or Hollywood screenwriters’’.^64^ Other reports not refuting the Western media narratives of the pandemic were mild and tend to praise the government for the ‘robust’ approach in handling the outbreak of the pandemic.

In *Le Monde,* choice of words was more fierce and unapologetic. For instance***, ***There is still little help, though, for the 5 million workers who are self-employed and those on zero-hours contracts: a slight raise to universal credit — a notoriously dysfunctional and discouraging benefits system…The allowances of a benefits system decimated by austerity will not suffice, and it shouldn’t have taken a deadly virus to make its shortcomings clear. The future looks precarious, too.^54^*CNN, NYT* and others also employed terms that changed with situations and events.

## Discussion

Scholarly evidence suggests that the masses form their opinions about public health crisis from the media.^68^ By extension, the way the media represents public health crisis such as the novel coronavirus goes a long way in determining how people react to it. Perhaps, this makes good the claim that ‘‘frames that paradigmatically dominate news are also believed to dominate audience’’.^69^ Our study supports this viewpoint that the global media framing of COVID-19 shapes the people’s response and understanding of the pandemic. It also aligns with Sandellet al^4^ who found that the nature of media framing of health information influences community’s perception and potential behaviour. In this study, human interest and fear/scaremongering represent the two dominant frames.

This finding aligns with the *constructionist* frame perspective. This paradigm assumes that ‘‘journalists are information processors who create ‘interpretative packages’ of the positions of politically invested ‘sponsors’ in order to both reflect and add to the ‘issue culture’ of the topic’’.^69^ The core message of the constructionist paradigm is that a given frame can retain dominance of coverage for a long time. In our subsequent research, we shall gauge this assertion to know the level of consistency or otherwise of the dominant frames found in this study. For now, our findings demonstrate that knowingly or unknowingly, media frames help people to construct reality about COVI9-19.

The preponderance of using fear/scaremongering might have accentuated fears in the public which may also lead to panic attack. This can aggravate the conditions of those with underlying health conditions. Indeed, analysis suggests a high rise in the number of panic attack in the UK due to the fear and anxiety propagated via different media platforms about the pandemic.^38^ One of the affected persons said, ‘‘you wake up, switch on the news, scroll through social media and see endless posts about coronavirus. Suddenly you’ve got a headache and your throat begins to feel dry. The symptoms are there when you’re worried but disappear when you’re distracted’’.^38^ It is therefore important for the media to prioritise only the vital and accurate information that would have minimal negative effects on the people. The people should also be re-oriented about making choices regarding what news to watch, read or listen to in order to minimise their level of exposure to news that has saturated the mediasphere about COVID-19. Using the right diction is also important in this regard to avoid stoking unnecessary fear among the public.

Our findings also demonstrate that in periods of crisis, management of information is crucial particularly the one that warrants immediate actions such as how to stay safe and prevent the spread of the pandemic. This becomes necessary because during a health crisis of international concern (such as the one caused by COVID-19), the public should be armed with access to genuine, crucial, accurate and current information to enable them make informed decision. It is therefore the responsibility of the media to ensure that news that would pollute the public domain is not given attention. As such, choice of words is important in crafting media reports to contain the advertent or inadvertent bombardment of the public with sensationalised, fake and inaccurate information caused by the pattern of frames adopted.

Huge differences exist regarding how the global media constructed hope and fear. In its obsession to keep churning out breaking news about COVID-19, the media may be doing more harm than good to public perception of the pandemic. This does not imply that the media should desist from breaking news about the pandemic, but the choice of words in doing this should tilt towards de-escalation of tension. Unlike Fogarty and colleagues^70^ which found that irrespective of the uncertainties surrounding the H1N1 pandemic, the television coverage was largely non-alarmist, our study demonstrates that the global media was generally alarmist especially by sensationalising the stories that further create fear and panic among the public. Conversely, Fogarty and colleagues^70^ argued that the Australian media acted responsibly by providing steady and highly transparent coverage of the H1N1 pandemic, but in our findings global media coverage was high, but lacks coherence and sufficient self-efficacy and this must have been caused by the obsession for breaking news across the media outfits.

The use of economic consequences frame underpins the importance of this element in weighing the economic impacts of the pandemic. Businesses around the world are crumbling and in the US, the hardest hit economy, more than 16% of the US workforce has filed for jobless benefits in a space of five weeks, the highest since the 2009 recession.^71^

The excessive use of attribution of responsibility (blame) frame may significantly shape people’s understanding of the pandemic and may result in more *post- ventive* than preventive measures as demonstrated in the coverage when US and European authorities spent so much time blaming China for the virus outbreak instead of working out preventive measures that would contain the outbreak before trying to understand whether the virus was a lab-made. This can also potentially spike racial discrimination and rift among countries. The claim that the virus was a bioweapon created in Wuhan lab has been vehemently denied, but that does not seem to exonerate China from blame, especially for allegedly hording vital information about the scale of the crisis during the early stage of the outbreak.^2^ For instance, information about the modes of transmission and preventive measures was limited at the onset of the pandemic. The Western media was also interrogated because attention was focused on blaming China while undermining the risks at home.

The tendency of reducing the COVID-19 pandemic to politicisation and ethnicisation risks polluting public response and behaviour towards the pandemic and may as well lead to escalation of tension while dissipating energy and resources that would have been employed to fight the virus.

### Limitation and recommendation for further studies

This study relied on published articles from the media, it did not draw from public response to such media messages. Therefore, there is a need to extend research enquiry into public response to address this gap. In-depth interviews and questionnaires can be used in this instance. Nonetheless, our study makes a robust assessment of the situation and should stimulate more thinking and understanding into the implication of the dominant media frames of the COVID-19 pandemic.

## Conclusion

This study finds a near identical framing pattern of COVID-19 among global media outfits analysed for this study. All of them employed words that accentuate fear among the people. It reinforces the relationship between media framing of health crisis and people’s perception and response to the risks of the virus. Human interest and fear/scaremongering dominated the coverage and this could potentially trigger more health challenges, especially for people with underlying health conditions. There is also a significant difference between how media framed hope and fear. In its obsession to keep churning out breaking news about COVID-19, the media may be causing information fatigue and influencing public perception of the pandemic. This does not imply that the media should desist from breaking news about the pandemic, but the choice of words in doing so should tilt towards de-escalation rather than escalation.

## Ethical approval

Our research focused on global media framing of COVID-19. The data for the study are in the public domain and also available in archives of the various media outfits we studied. The study did not involve human subjects. Nonetheless, our study adheres strictly to the research guidelines of Ebonyi State University, Abakaliki. As such, ethical approval for this study was secured from the Department of Mass Communication’s Ethical Committee with the number: EBSU/MAC/EC/20/0051.

## Competing interests

The authors declare no conflict of interests.

## Funding

None.

## Authors’ contributions

JNO, ECO, JC sourced and wrote the literature in addition to analysing the data. CJN, ESN, SUN and SCN sourced and harmonised the data for analysis. While SE and NIO coded the data. JNO and JC helped in putting the content analysis in perspective as well as editing the final draft.

## Disclaimer

The data used for this analysis are available in the public domain and can be accessed by anybody from the archives of the media organisations we studied. Although the data were useful for our analysis, we do not make representation or warranty regarding the accuracy, adequacy, validity, and reliability of the opinions shared by these media outfits. Our study adheres strictly to the fair use policy of the materials used in accordance with the provisions of section 107 of the US copyright law which permits any non-commercial sharing with attribution.

## Acknowledgments

We acknowledge Dr Emmanuel Chike Onwe for providing us with his office space where we regularly brainstormed before, during and after the research.

**Table 1:** Frames adopted for the study and their examples in the reported COVID-19 stories

**Frame Categories**	**Definition**	**Example**
Economic consequences	In this frame, issues relating to event being reported are approached from the perspective of their economic consequences on the individuals, organisations, or country.	‘‘As stock markets plummeted in March, shares in pharmaceutical company Gilead Sciences rose by 20% on the news of clinical trials of their antiviral remdesivir for the treatment of Covid-19’’. ^20^
Human interest	Semetko and Valkenburg^18^ explain that journalists give ‘‘a human face or an emotional angle to the presentation of an event, issue, or problem’’	‘‘Mother dies from coronavirus hours after giving birth to her first child in Ukrainian hospital’’.^21^
Conflict	Semetko and Valkenburg^18^ explain that this frame ‘emphasizes conflict between individuals, groups, or institutions as a means of capturing audience interest’	‘‘New York ICU nurse: Battling coronavirus feels 'like a war'".^22^
Morality/Religion	Semetko and Valkenburg^18^ note that morality frame ‘‘puts the event, problem, or issue in the context of religious tenets or moral prescriptions’’.	‘‘As concern over the spread of coronavirus grows, people around the world are changing the way they do things. Churches, mosques, temples and synagogues are also changing rituals in an effort to contain the spread of the virus’’.^23^
Attribution of Responsibility	Semetko and Valkenburg^18^ explain that this frame ‘‘presents an issue or problem in such a way as to attribute responsibility for its cause or solution to either the government or to an individual or group’’.	New York's coronavirus outbreak came from Europe and other parts of US.^24^
Politicisation	Politicisation frame is noticed when attacks by the insurgents are reported and interpreted from political perspectives. It is politicisation when such frames wear the toga and colouration of politics, especially.^19^	"Accusations won't get rid of the virus" "Smearing and accusations won't get rid of the virus," said Chinese Foreign Ministry spokesperson Zhao Lijian at a press conference in Beijing.^25^
Ethnicisation	This frame foregrounds ethnic terms while interpreting stories.^19^	‘‘French doctors are accused of racism and 'treating Africans like human guinea pigs' after saying Covid-19 vaccines should be trialed there’’.^26^
Fear/Scaremongering	Stories that are exaggerated to cause fear or panic among the public.	Chinese President Xi Jinping has warned against the risk of a second wave of infections in the country as the global pandemic continues to spread.^27^
Hope	In this frame, emphasis shifts to stories that give people hope and reassure them even in the midst of the crisis.	‘‘Our world has changed immensely in the last few weeks but amid the upheaval and distress, there are reasons to believe we can emerge from the crisis with some human qualities enhanced’’.^28^

**Table 2:** Dominant frames used in the coverage of COVID-19

**Frame**	**Variable**	**Media**								**Total** **No. (%)**
		**BBC** **No. (%)**	**Daily Mail** **No. (%)**	**Le Monde No. (%)**	**Vatican** **No. (%)**	**CNN** **No. (%)**	**NYT** **No. (%)**	**Punch** **No. (%)**	**Pples Daily** **No. (%)**	
Economic Conseq	Loss	112 (10.9)	96 (8.5)	31 (8.9)	-	101 (9.7)	56 (5.7)	34 (7.4)	71 (9.6)	502 (8.1)
	Gain	21 (2.1)	32 (2.8)	10 (2.9)	-	57 (5.5)	46 (4.7)	20 (4.4)	23 (3.1)	208 (3.4)
Human interest	Positive	76 (7.4)	103 (9.2)	17 (4.9)	35 (8.1)	37 (3.6)	101 (10.3)	68 (14.8)	49 (6.6)	479 (7.7)
	Negative	296 (28.9	307 (27.3	48 (13.8	47 (10.9)	123 (11.8)	191 (19.5)	22 (4.8)	11 (1.5)	1040 (16.9)
Conflict	Conflict	32 (3.1)	31 (2.8)	06 (1.8)	-	135 (12.9)	144 (14.6)	02 (0.4)	25 (3.4)	375 (6.2)
Morality/ Religion	Positive	18 (1.8)	14 (1.2)	-	26 (6.0)	44 (4.2)	19 (1.9)	12 (2.6)	35 (4.7)	163 (2.6)
	Negative	51 (4.9)	31 (2.8)	-	98 (22.6)	85 (8.2)	11 (1.1)	53 (11.6)	06 (0.8)	354 (5.8)
Att of Res	Blame	88 (8.6)	105 (9.3)	78 (22.5)	-	118 (11.3)	83 (8.4)	78 (17.0)	88 (11.9)	638 (10.4)
Politicisation	Blame	27 (2.6)	36 (3.2)	35 (10.1	14 (3.2)	76 (7.3)	69 (7.0)	17 (3.7)	78 (10.6)	350 (5.7)
	Defence	-	16 (1.4)	-	-	12 (1.2)	07 (0.7)	04 (0.9)	118 (16.0	157 (2.6)
Ethnicisation	Positive	21 (2.1)	17 (1.5)	11 (3.2)	11 (2.5)	23 (2.2)	22 (2.2)	10 (2.1)	11 (1.5)	125 (2.0)
	Negative	09 (0.9)	05 (0.4)	-	-	44 (4.2)	13 (1.3)	02 (0.4)	40 (5.4)	114 (1.9)
Fear/Scaremongeri	Positive	101 (9.9)	151 (13.4	54 (15.6)	09 (2.1)	44 (4.2)	117 (11.9)	75 (16.4)	31 (4.2)	582 (9.5)
	Negative	21 (2.1)	34 (3.0)	11 (3.2)	36 (8.3)	67 (6.4)	67 (6.8)	08 (1.7)	65 (8.8)	309 (5.0)
Hope	Solution	59 (5.8)	44 (3.9)	25 (7.2)	94 (21.7)	24 (2.3)	14 (1.4)	36 (7.7)	54 (7.3)	354 (5.8)
	Palliative	91 (8.9)	102 (9.0)	21 (6.1)	63 (14.5)	51 (4.9)	22 (2.2)	17 (3.7)	32 (4.3)	395 (6.4)
**Total**		**1023 (16.6)**	**1124 (18.3)**	**347 (5.6)**	**433 (7.0)**	**1041 (16.9)**	**982 (15.9)**	**458 (7.5)**	**737 (11.9**	6145 **(100)**
